# Photocatalysis of Methyl Orange (MO), Orange G (OG), Rhodamine B (RhB), Violet and Methylene Blue (MB) Under Natural Sunlight by Ba-Doped BiFeO_3_ Thin Films

**DOI:** 10.3390/ma18040887

**Published:** 2025-02-18

**Authors:** Abderrahmane Boughelout, Abdelmadjid Khiat, Roberto Macaluso

**Affiliations:** 1 Laboratory of Material Physics, Faculty of Physics, University of Sciences and Technology-Houari Bouediene (U.S.T.H.B.), El-Alia, Bab Ezzouar, P.O. Box 32, Algiers DZ-16111, Algeria; 2 Research Center in Industrial Technologies (CRTI), Cheraga, P.O. Box 64, Algiers DZ-16014, Algeria; khiatmadjid2018@gmail.com; 3 National Research Centre for Biotechnology, P.O. Box E73, Constantine DZ-25000, Algeria; 4 Thin Films Laboratory (TFL), Dipartimento di Ingegneria, Università degli Studi di Palermo, Viale delle Scienze (Ed. 9), 90128 Palermo, Italy

**Keywords:** perovskite films, BFO, BBFO2, optical properties, photocatalytic activity, RhB, OG, MO, violet, MB

## Abstract

We present structural, morphological, optical and photocatalytic properties of multiferroic Bi_0.98_Ba_0.02_FeO_3_ (BBFO2) perovskite thin films prepared by a combined sol–gel and spin-coating method. X-ray diffraction (XRD) analysis revealed that all the perovskite films consisted of the stable polycrystalline rhombohedral phase structure (space group *R3c*) with a tolerance factor of 0.892. By using Rietveld refinement of diffractogram XRD data, crystallographic parameters, such as bond angle, bond length, atom position, unit cell parameters, and electron density measurements were computed. Scanning electron microscopy (SEM) allowed us to assess the homogeneous and smooth surface morphology of the films with a small degree of porosity, while chemical surface composition characterization by X-ray photoelectron spectroscopy (XPS) showed the presence of Bi, Fe, O and the doping element Ba. Absorption measurements allowed us to determine the energy band gap of the films, while photoluminescence measurements have shown the presence of oxygen vacancies, which are responsible for the enhanced photocatalytic activity of the material. Photocatalytic degradation experiments of Methylene Blue (MB), Methyl orange (MO), orange G (OG), Violet and Rhodamine B (RhB) performed on top of BBFO2 thin films under solar light showed the degradation of all pollutants in varying discoloration efficiencies, ranging from 81% (RhB) to 54% (OG), 53% (Violet), 47% (MO) and 43% (MB).

## 1. Introduction

In recent years, perovskite-type oxides have gained considerable attention for their diverse and intriguing physical properties. Among these compounds, bismuth ferrite (BiFeO_3_), commonly referred to as BFO, has been the subject of extensive research due to its multiferroic [1,2,3] and photocatalytic nature [4]. The BFO’s unit cell belongs to the *R3c* space group and exhibits an ABO_3_ type of rhombohedral distorted perovskite structure [5]. The material’s high Curie temperature (TC, approximately 1103 K) and Neel temperature (TN, about 643 K) make it an appealing choice for practical applications, such as in transducers, new actuators, sensors, capacitive/inductive passive filters for communications, and magnetic field probes, to name some. Recently, these systems have gained significant attention due to their unique features and multifunctional characteristics [6,7,8], including the efficiency of BiFeO_3_ thin films for pollutant degradation [9,10,11]. Photocatalysis is a consolidated technology for degrading pollutants that are present in water stemming from large industrial facilities. Its effectiveness in degrading a wide range of pollutants in water, including those of an industrial origin, makes it a highly versatile tool for environmental cleanup. Furthermore, compared to other traditional methods, it appears to be a low-cost technique as it uses sunlight as an energy source and has minimal environmental impact. In fact, unlike traditional methods that rely on harsh chemicals which generate harmful byproducts, photocatalysis breaks down pollutants into harmless molecules like water and carbon dioxide. This combination of effectiveness, affordability, and environmental friendliness positions photocatalysis as a frontrunner technology in the ongoing quest for innovative water treatment solutions. While challenges remain in optimizing catalyst efficiency and scaling the technology for large-scale applications, the potential benefits are undeniable, propelling photocatalysis to the forefront of research and development efforts [11,12,13,14].

To improve the anti-ferromagnetic and photocatalytic properties of BFO, researchers have widely adopted the doping method [12,13,14]. Doping at the A site influences the centrosymmetric nature of the FeO_6_ octahedral structure and creates both oxygen vacancies, and multiple valence states of the Fe ion, which may contribute to improved and accelerating photocatalytic properties of the BFO [15]. These vacancies play, in fact, a role as active sites on the surface and generate further •OH radicals, which act as active centers to capture photo-induced electrons, accelerating and improving the photocatalytic activity of the material because of the simultaneous inhibition of the recombination of photo-induced electrons and holes [16,17,18,19].

Additionally, the variable oxidation states of Fe ions, ranging from Fe^3+^ to Fe^2+^, result in altered multiferroic properties in BFO [17]. Barium is widely studied as a dopant for BFO to improve its multiferroic properties. By varying the amount of Ba, it is possible to obtain Bi_0.98_Ba_0.02_FeO_3_ (BBFO2) [20]. The reasons for using barium as a dopant of BFO are twofold: (i) BFO’s structure includes Bi ions in 3^+^ oxidation states, while Ba ions exist in 2^+^ oxidation states. By replacing Bi with Ba, oxygen vacancies can be created in BFO, which can impact the magnetic and photocatalytic behavior of the film; (ii) Ba^2+^ has a larger ionic radius (1.35 Å) than Bi^3+^ (1.03 Å), which alters the Fe-O-Fe bond angle and affects the magnetic behavior. Khomchenko et al. have investigated the effect of Ba, Ca, Sr, and Pb doping at the A site of BFO. They found that the cell parameters and magnetization are proportional to the ionic radius of the dopant [21]. Different synthesis techniques, including solid-state reactions for BFO powder preparation to be used for thin-film deposition by pulsed laser deposition (PLD) [21,22], the sol–gel method [23] and mechanically supported synthesis [24], have been employed for obtaining doped and non-doped BFO films. Among these techniques, sol–gel-assisted spin coating is an attractive, easy and inexpensive method which has the advantage of obtaining thin films with the desired stoichiometry [4,23].

In this work, based on the photocatalytic properties of Bi_1−x_Ba_x_FeO_3_ thin films for Rhodamine (B) (RhB) reported in our previous work [4], we aim to present the ability of Ba-doped BFO thin films to degrade, under natural sunlight, a wider number of pollutants, such as Methylene Blue (MB), Methyl orange (MO), orange G (OG) and Violet. Rhodamine (B) degradation studies are also included for comparison.

Structural, morphological and optical properties of BBFO2 thin films are also reported.

## 2. Materials and Methods

BBFO2 thin films were synthesized using a sol–gel-assisted spin-coating technique on 2 cm × 2 cm glass substrates, as reported in [4]. Bismuth nitrate pentahydrate (Bi(NO_3_)_3_·5H_2_O), iron nitrate nonahydrate (Fe(NO_3_)_3_·9H_2_O), and barium acetate ((CH_3_COO)_2_Ba) were employed as initial precursors and dissolved in ethylene glycol as a solvent to produce a final precursor solution with a concentration of 0.2 M. In order to produce a well-mixed BBFO2 gel solution, the resulting solutions were mixed and then stirred for one hour at 80 °C. A few drops of acetic acid were added to the BBFO2 solution to act as stabilizer. Following that, the obtained solution was spin-coated onto clean glass substrates (3000 rpm for 40 s) to produce uniform films which underwent, afterwards, a drying treatment at 320 °C for five minutes. Five iterations of the spin coating and drying treatment procedures were carried out. All samples were, finally, annealed at 500 °C for four hours in an ambient atmosphere to fully crystallize and consolidate the films.

Structural and phase formation properties of the BBFO2 thin films after the thermal treatment step were checked at room temperature with a PANalytical Empyrean X-ray (Almelo, Netherlands) diffractometer using CuKα radiation (λ = 1.5406 Å) and operating in the 2θ range of 20°–70°, with a step size of 2θ = 0.02°. The phase identification was performed using X’pert High Score software (version 4.9) supported with the ICDD database. Rietveld refinement was performed on the X-ray patterns obtained using the FullProf computer program (version 5.0) [25]. In this way, lattice parameters, atomic coordinates, thermal parameters, occupancies, and microstructural parameters were refined for all the samples. In the structural analysis, an asymmetric Thompson–Cox–Hastings pseudo-Voigt function was used to describe the peak shapes for different samples. The GFourier program (FullProf package) was used for the visualization of electron density within the unit cell. The films’ morphology was examined by using a scanning electron microscope (SEM, type JEOL JCM-5000 Neoscope, JEOL Ltd., Akishima, Japan). UV–visible absorption spectra of the films were recorded by using a UV-Vis spectrophotometer (Lambda 35, from Perkin Elmer, Waltham, MA, USA), while photoluminescence (PL) spectra were observed at room temperature using a 325 nm laser excitation source.

**Figure 1:**
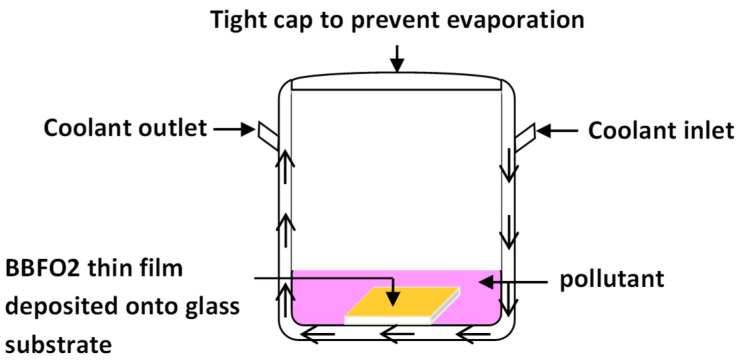
Schematic diagram of the reactor used for the photocatalytic experiments. The arrows indicate the direction of the coolant.

The photocatalytic degradation of Methylene Blue (MB), Methyl orange (MO), orange G (OG), Violet and Rhodamine (B) (RhB) by BBFO2 thin films was performed by exposing the films to natural sunlight with an average radiative flux of about 800 mW/cm^−2^ for 6 h. BBFO_2_ thin films were placed into a Pyrex reactor container, previously filled with the pollutant solution. A schematic of the reactor is reported in Figure 1. In order to maintain a constant temperature of 25 °C, the reactor was placed for cooling into a thermostatic water bath during the measurements. All the experiments were carried out during clear sunny days of May, at the same hours of the day. The radiation intensity was measured at the beginning and at the end of each experiment. In each experiment, the solution for each pollutant (MB, MO, OG, RhB and Violet, 100 mL volume, 10 mg/L concentration) was taken for batch studies [4,26].

## 3. Results and Discussion

### 3.1. Phase, Structural, and Morphological Characterization

**Figure 2:**
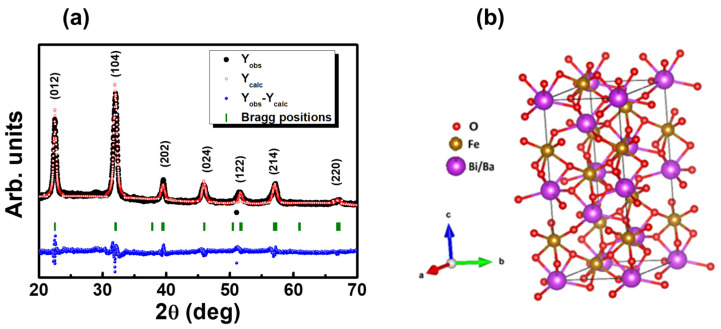
(**a**) Measured (black), Rietveld-refined (red), and difference between measured and refined data (blue) XRD patterns using the FullProf program for a BBFO2 thin film. The vertical bars (green) indicate the angular position of the allowed Bragg reflections. (**b**) The three-dimensional schematic representation of the BBFO2 unit cell with a trigonal structure in a hexagonal setting.

The X-ray diffraction (XRD) pattern, recorded at room temperature, and the Rietveld analysis conducted using the FULLPROF software are presented in Figure 2a. XRD results revealed that the prepared BBFO2 thin films exhibit a single-phase rhombohedral perovskite structure (*R3c* space group) as determined by ICDD Ref. Code No. 01-082-1254, with no discernible secondary phases within the experimental limits of the XRD analysis. As it is possible to see, a good level of agreement between the observed (black circles) and the calculated (red open circles) XRD patterns was achieved for the exanimated Bi_0.98_Ba_0.02_FeO_3_ film.

The stability of perovskite compounds is usually described in terms of Goldschmidt’s tolerance factor (*t*). This is an indicator of the geometric stability and distortion of the crystal structure in terms of the constituent ionic packing [27]. In our case (perovskite with an ABO_3_ structure), it can provide information about the Bi_1−x_Ba_x_FeO_3_ (x = 0.02) structure stability after Ba doping. For Bi_1−x_Ba_x_FeO_3_, Goldschmidt’s tolerance factor can be written as [28]:

(1) $$t=\frac{\left(\left(1-x\right)r_{Bi}+xr_{Ba}\right)+r_{0}}{\sqrt{2}\left(r_{Fe}+r_{0}\right)}$$

where *r_Bi_, r_Ba_, r_Fe_* and *r_0_* are the effective ionic radii of Bi, Ba, Fe and O ions, respectively. The ideal tolerance factor (i.e., for ideal cubic perovskite structure) is 1, but when the ratio of the ionic radii deviates from the ideal value (*t* ≠ 1), a geometric strain and crystal distortions arise. By using Shannon ionic radii, pure BFO has been found with a tolerance factor of about 0.89 [29]. Calculations, carried out by using tabular ionic radii, allowed us to obtain a tolerance factor *t* = 0.892 for Bi_0.98_Ba_0.02_FeO_3_, which is very close to the tolerance factor of the undoped BiFeO_3_ film (*t* = 0.89). This means that Ba doping has not considerably modified the structure stability of the material.

Crystallite size (*D_hkl_*) was calculated using Scherrer’s equation [30]:

(2) $$D_{hkl}=K\lambda /\beta_{hkl}\cos\theta$$

where *K* is the factor related to crystallite (best possible value of the factor = 0.89), *β_hkl_* is the full width at half maximum (FWHM) of the considered peak intensity, *θ* is the Bragg’s diffraction angle, and *λ* is the wavelength of the used radiation (*λ* = 0.15405 nm). The crystallite size of the (104) plane at a diffraction angle of 32° is 14.55 nm.

Cell parameters were used to obtain, through the VESTA program (version 3.5.8) [31], the schematic representation of the BBFO2 structure reported in Figure 2b, which shows that the BBFO2 perovskite has a symmetrical structure made of staggered planar BiO_6_ and FeO_6_ complexes that construct the *R3c* space group.

**Figure 3:**
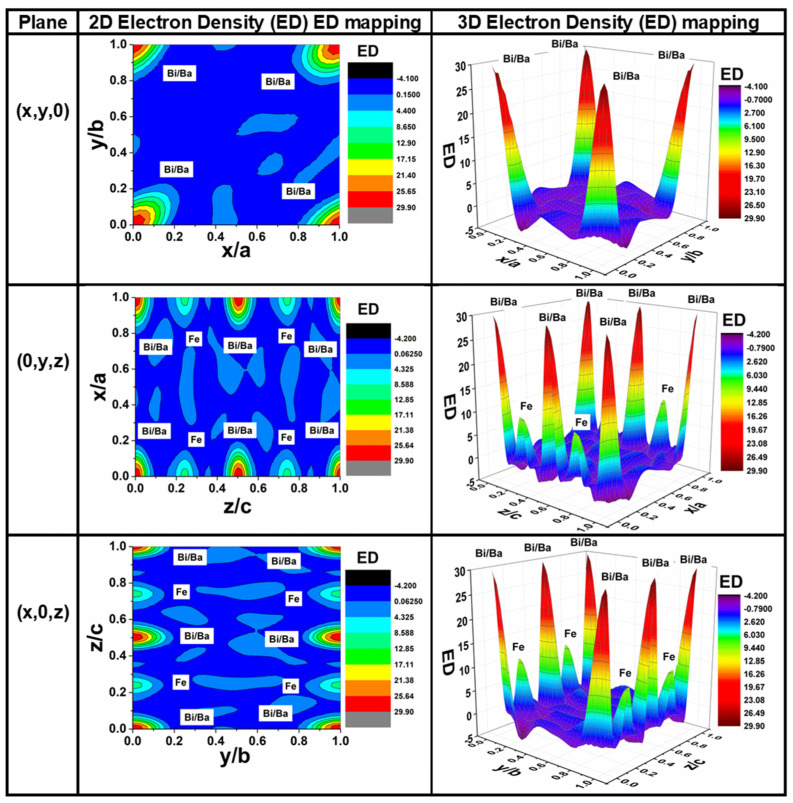
Two-dimensional and three-dimensional Fourier maps along (x, y, 0), (0, y, z) and (x, 0, z) planes to visualize the electron density (ED) distribution for the BBFO2 film, measured in the number of electrons per cubic Angstrom, n/Å^3^.

In order to understand and confirm the structural distortion in the localized environment, the electron density distribution around atoms was mapped using the GFourier 04.06 program of FullProf software [32]. The 3D and 2D (contours) Fourier maps along (x, y, 0), (0, y, z) and (x, 0, z) visualize the distribution of electron density in the BBFO2 unit cell (Figure 3). The electronic charge density map of the BBFO2 film shows strong charge localization along the radius of Bi and Ba atoms interacting with the adjacent O atoms, which confirms the prevalence of the ionic character of the single Ba-O and Bi-O bonds [29,30,31,32,33,34,35]. This ionic bond results from a large difference in Pauling electronegativity between O and Fe and the elements Ba and Bi [36]. Because of the larger atomic number of Ba and Bi atoms (Ba (Z = 56); Bi (Z = 83)) compared to the Fe atom (Fe (Z = 26)) [37], the maximum electron density (∼29 n/Å^3^) was found for Bi/Ba atoms along to the xy, xz and yz direction, while the electron density due to Fe atoms was ~12 n/Å^3^ along the xz and yz direction, as illustrated in Figure 3. We can also conclude that the electronic density map along the three planes, xy, xz and yz, confirms the hexagonal crystal structure, the crystal symmetry (*R3c* space group) and the atoms’ position illustrated in Table 1.

The pseudo-Voigt function was utilized to refine the XRD peak shape [38]. Moreover, zero shift, background, atomic positions, specimen displacement, lattice parameters, preferred orientation, FWHM, anisotropic temperature parameters, transparency, scale factor and shape parameters were varied during the refinement process [39]. The information presented in Table 2 includes the diffractometer parameters employed for data collection, the crystal structure parameters derived from simulation, and the reliability factors. The observed distance between Bi/Ba and Fe with O and atom position are reported in Table 1.

**Figure 4:**
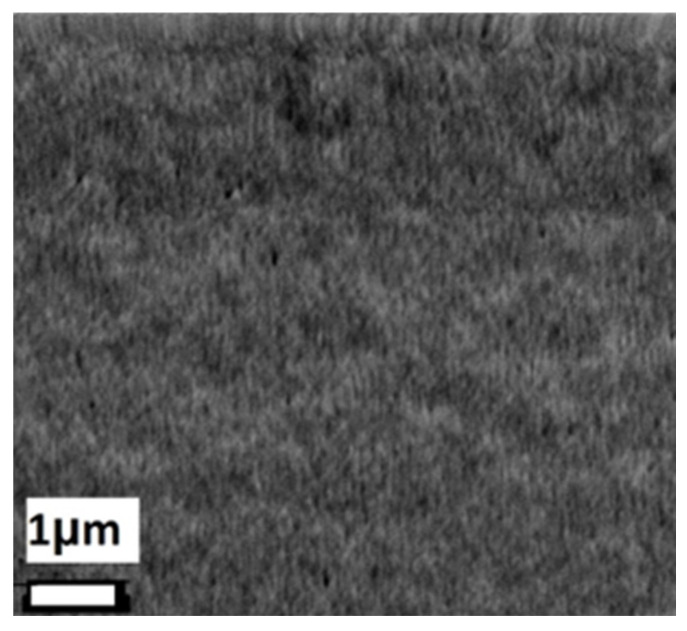
SEM top view of a representative BBFO2 thin film.

Figure 4 shows the surface SEM image of a representative BBFO2 thin film; it can be observed that the film presents a small degree of porosity with a smooth surface morphology.

### 3.2. Chemical Surface Composition Characterization

**Figure 5:**
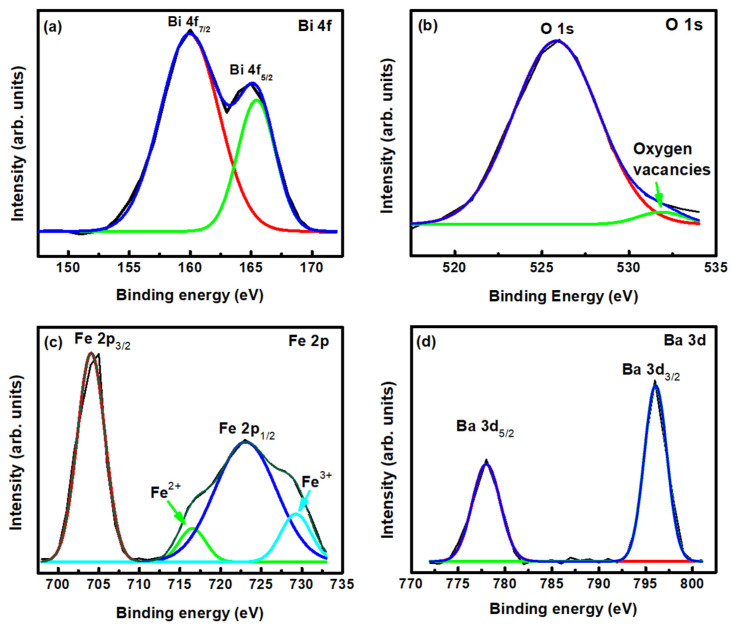
Deconvoluted core level XPS spectra of (**a**) Bi 4f, (**b**) O 1s, (**c**) Fe 2p and (**d**) Ba 3d of a Ba-doped BFO thin film. The black curves represent the experimental data, while the blue curves are the corresponding fittings. Red, green and cyan curves are the fitted subpeaks.

In order to obtain more information, including defects, on the surface chemical composition of BBFO2 films, X-ray photoelectron spectroscopy (XPS) measurements were carried out. Results are shown in Figure 5 and reveal the presence of Bi, Fe, O and the doping element Ba. The spectrum of the Bi element is reported in Figure 5a. It clearly shows the presence of two characteristic peaks at 159 eV and 165 eV, which can be ascribed to Bi 4f_7/2_ and Bi 4f_5/2_, respectively. These binding energy values are compatible with the Bi^3+^ oxidation state, corresponding to Bi_2_O_3_ [40], which reflects a dominant Bi^3+^ oxidation. The oxygen spectrum, reported in Figure 5b, can be fitted by two Gaussian peaks. The first one, at 525 eV (red curve), corresponds to the characteristic of lattice oxygen–metal bonds, while the second one, at 531 eV (green curve), can be ascribed to the presence of defects associated with the oxygen element, namely oxygen vacancies and adsorbed oxygen at the surface of the samples [40]. Figure 5c highlights the presence of the Fe^3+^ oxidation state, with the peaks located at the binding energy of 705 eV and 723 eV, corresponding to the Fe 2p_3/2_ and 2p_1/2_ components, respectively [41]. These peaks can be due to the spin–orbit coupling interaction. In addition to the main peaks, two satellite peaks are observed at 716.52 eV and 729.26 eV confirming the presence of the Fe^3+^ oxidation state in these ceramics. The component peaks appeared due to the deconvolution of the Gaussian curve fit of the first main peak indicating the presence of Fe^2+^ and Fe^3+^ oxidation states. The XPS spectrum related to the doping element (Ba), is reported in Figure 5d. It shows two peaks at 778 eV and 796 eV, corresponding to Ba 3d_5/2_ and Ba 3d_3/2_, respectively. These peaks indicate that the Ba atoms are in the Ba^3+^ oxidation state.

### 3.3. Optical Characterization

**Figure 6:**
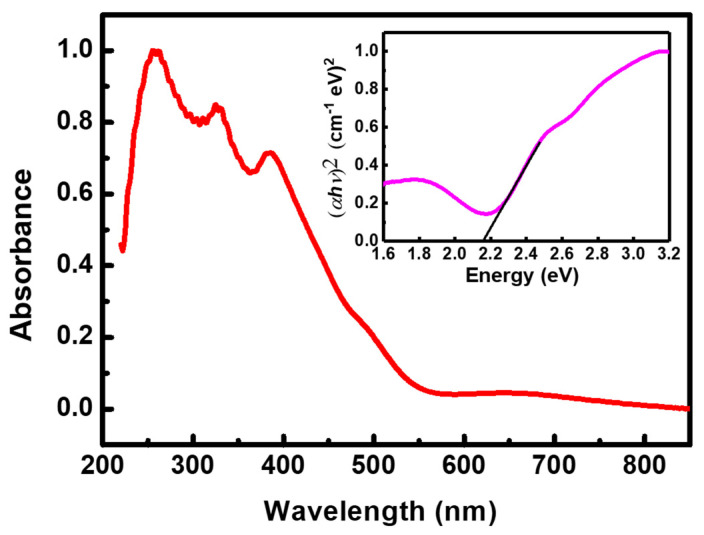
BBFO2 film absorbance spectrum. The inset shows Tauc’s plot for energy band gap determination.

The optical characteristics of the fabricated BBFO2 films were evaluated by recording their UV-Vis absorption spectra and their photoluminescence (PL) at room temperature. The first offer significant insights into the electronic states of the material since the absorption edge in the UV-Vis range is closely related to the band gap energy of the photocatalyst [42]. In Figure 6 is reported the absorption spectrum of a representative BFFO2 film. As it is possible to see, the absorption range is approximately 240–540 nm, indicating the potentiality of BFFO2 films in UV–visible light photocatalysis. To estimate the energy band gap of the films, Tauc’s formula [4], given by the following equation, was used:

(3) $$\alpha h\nu =A{\left(h\nu -E_{g}\right)}^{n/2}$$

where *α*, *h*, *ν*, *E_g_*, *A*, and *n* represent the absorption coefficient, the Planck constant, the light frequency, the energy band gap, the proportional constant, and an index depending on the nature of the transition, respectively. Assuming that *n* = 1, considering that BiFeO_3_ is a direct band gap material [42], an energy gap of 2.16 eV can be estimated by extrapolating the straight line portion of the curve at *α* = 0 (see inset of Figure 6). This value is smaller than the energy gap determined for the undoped BFO film [4] and anticipates a possible beneficial impact to sunlight-performed photocatalytic experiments.

**Figure 7:**
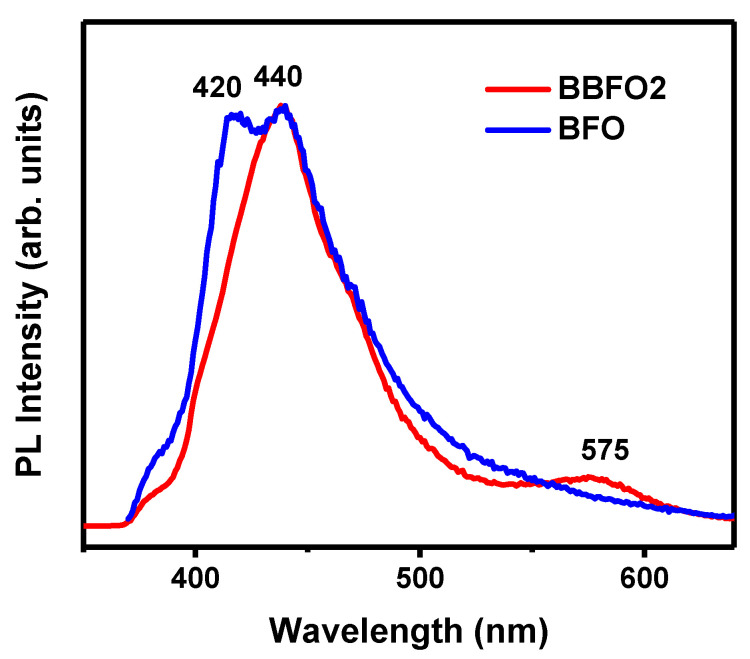
Photoluminescence spectrum of undoped (blue curve) and Ba-doped (red curve) BFO thin films.

Photoluminescence spectroscopy is one of the most important tools to gather information about the electronic structure of a material, including also intraband energy levels that are ascribable to defects within the material [24,25]. Moreover, the interface charge carrier transfer process can be studied [43]. In Figure 7 is reported the PL spectrum of a BBFO2 film (red curve), recorded at room temperature in the 350–640 nm range (excitation source 325 nm). The undoped BFO film PL spectrum (blue curve) is also reported as a reference. It displays, similarly to other reported works [44], two peaks centered at 420 nm and 440 nm that are ascribable to the band-to-band electronic transition in BFO (electron transition from the conduction band to the valence band) [28,29,30,31,44].

The doped film (BBFO2) shows instead a broad peak centered at 440 nm (2.82 eV), due to the characteristic band-to-band transition of BFO, and a broad emission at around 575 nm that is ascribable to defect levels (e.g., due to oxygen vacancies formed into the doped film) which may serve as alternative pathways for electronic recombination [45,46].

The absence of near-band gap transitions is a strong indication of the high crystallinity of the deposited film. This is consistent with the results of the XRD characterization, which showed that the synthetized BBFO2 films have a well-ordered crystalline structure.

### 3.4. Photocatalysis Experiments

**Figure 8:**
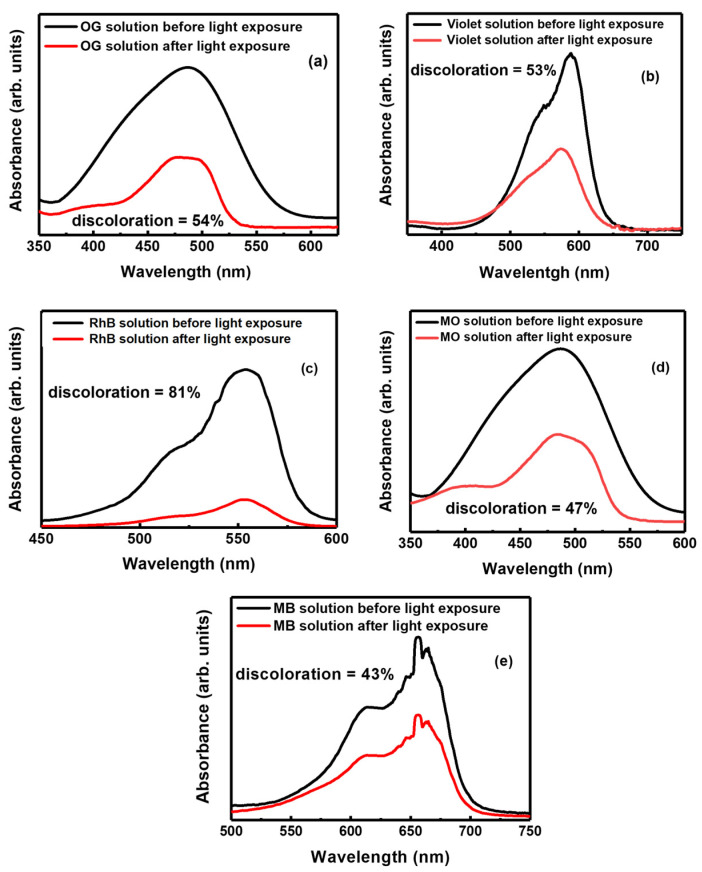
Absorption spectra before and after the photodegradation of (**a**) OG, (**b**) Violet, (**c**) RhB, (**d**) MO and (**e**) MB solutions in the presence of BBFO2 films before and after light exposure (6 h). Each plot reports the degradation percentual with respect to the non-exposure condition.

Photocatalytic degradation of MO, OG, MB, RhB and Violet was performed by exposing Ba-doped BFO thin films combined with the correspondent pollutant solution to solar light for 6 h. The aqueous solution was chosen to have the pH value of about 7. The corresponding absorption spectra were recorded in the 350–700 nm wavelength range [4,34]. Additionally, for each degradation test, a reference absorption measurement was carried out in the dark to account for any degradation that may have occurred due to other factors. Results, reported in Figure 8, show that each pollutant is degraded with different efficiencies. In particular, RhB is the most degraded pollutant (81%), while MB undergoes the smallest degradation (43%), which is however comparable to what was reported in [47,48], where BiFeO_3_/CuWO_4_ and Bi_2_O_3_/Bi_2_O_2_CO_3_ heterojunctions are used as catalysts, respectively.

The origin of the observed photocatalytic activity of the Ba-doped BFO thin films for dyes’ degradation could be ascribed to the formation of surface oxygen vacancies revealed both by XPS and optical measurements. Oxygen vacancies act as trapping centers for photoinduced electrons, thus facilitating the separation of the photogenerated electron-hole pairs as well as the production of active species (hydroxyl radicals) for an enhanced photodegradation of the analyzed dyes (RhB, MB, MO, OG, and Violet) [15,16,18]. In addition, they suppress the recombination of photogenerated electrons and holes favoring the adsorption of dyes molecules.

**Figure 9:**
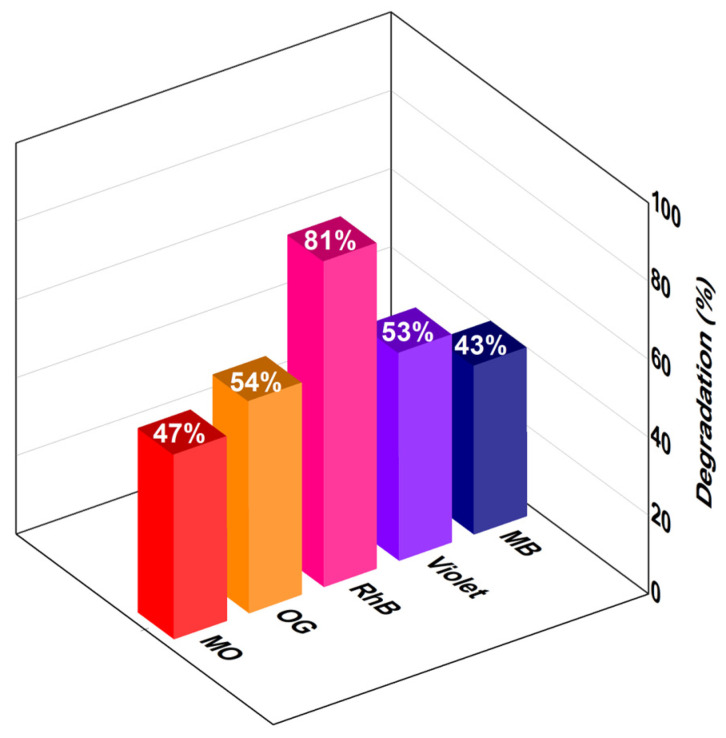
Photocatalytic degradation of MO, OG, RhB, Violet and MB.

**Figure 10:**
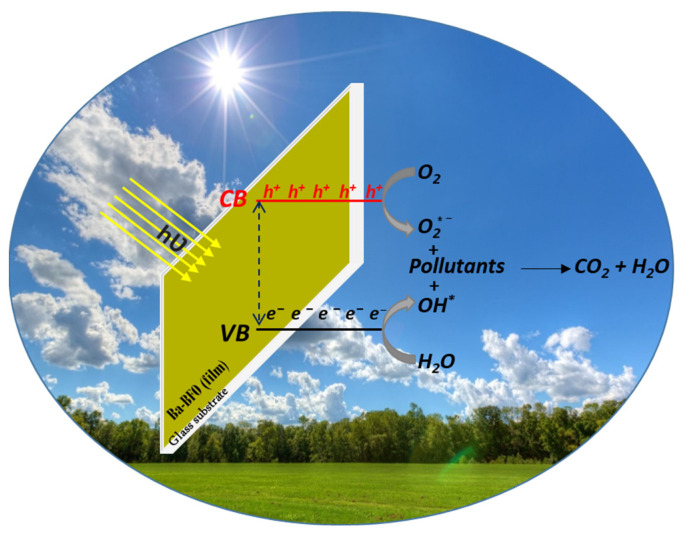
Photocatalytic mechanism diagram of MO, OG, RhB, Violet and MB in Ba-doped BFO thin films, under natural sunlight.

The variation in photocatalytic degradation for the different pollutants highlighted in Figure 9 can be attributed to both the structural properties of thin films and the pollutant. For example, it has been reported in previous works [43] that the photocatalysis was higher for pollutants whose absorption energy is close or coincident with the energy levels present in the used thin films. These levels may be a source of electrons that contribute to the photocatalysis process, which increases the effectiveness in photodegradation of these pollutants [34,41]. This is because the photocatalytic process depends primarily on the concentration of charges that can be excited by sunlight, which results in generated electron-hole pairs (*e^−^-h^+^*) on the surface of the BBFO2 thin films (Figure 10), following the relationship:

Semiconductor (BBFO2) + *hν* → *e^−^ + h^+^* (4)

The latter (*e^−^* and *h^+^*) are involved in the photocatalytic process of the target pollutants based on the following equations [25,34,35,36]:

(5) $$e^{-}+O_{2}\;\to \;O_{2}^{*-}$$

(6) $$h^{+}+H_{2}O\;\to \;{OH}^{*-}+H^{+}$$

(7) $$h^{+}+{\mathrm{OH}}^{-}\;\to \;{OH}^{*}$$

(8) $$\mathrm{Pollutants}+\mathrm{radicals}\;({OH}^{*}\;\mathrm{or}\;O_{2}^{*-})\;\to \;\mathrm{Degradation}\mathrm{products}\;(e.g.,\;{CO}_{2},\;H_{2}O)$$

A comparison with photocatalytic experiments performed by employing different catalysts shows a smaller degradation of RhB (60%) with the Ni-doped ZnO catalyst [49], which is enhanced to 94% by using a photoelectrocatalytic removal process on the BiWO_6_ catalyst [50]. On the other hand, the almost complete photodegradation of OG (98%) [51], Violet (99%) [52], and MO (95%) [53] has been achieved by using Bi_2_MoO_6_, NiO, and Fe-doped ZnO as catalysts, respectively.

## 4. Conclusions

Multiferroic Bi_0.98_Ba_0.02_FeO_3_ perovskite thin films have been successfully synthesized through sol–gel and spin-coating. XRD analysis at room temperature confirms the single-phase character of BBFO2 thin films with a rhombohedral structure perovskite with space group *R3c* (No. 62), without any impurity phase, indicating that barium atoms are well substituted. The tolerance factor confirmed the phase stability of the samples, and the crystallite size calculated using Scherrer’s equation is found to be 14.55 nm. XPS measurements also showed, together with the presence of Bi, Fe, O and the doping element Ba, the presence of oxygen vacancies and adsorbed oxygen at the surface of the samples, while SEM characterization indicated the smooth surface morphology of the films. UV-Vis absorption measurements showed the good potentiality of BFFO2 films in UV–visible light photocatalysis and allowed us to estimate an energy band gap of about 2.16 eV, which is slightly smaller than the value of BiFeO_3_ thin films reported in the literature (about 2.3 eV) [54].

Photoluminescence measurements have shown a broad peak centered at 440 nm, which can be associated with the characteristic band-to-band electronic transition observed in BFO films, and a broad emission at around 575 nm that is ascribable to oxygen vacancies which may serve as alternative pathways for electronic recombination.

The experiments of photocatalytic degradation of MO, OG, MB, RhB and Violet by Ba-doped BFO thin films under solar light showed different degradations for each pollutant, with RhB reporting the largest photodegradation (81%), followed by OG (54%), Violet (53%), MO (47%) and MB (43%). The origin of the observed photocatalytic activity of BBFO2 thin films could be ascribed to the formation of surface oxygen vacancies which, on one hand, act as trapping centers for photoinduced electrons, thus facilitating the separation of the photogenerated electron-hole pairs as well as the production of hydroxyl radicals for an enhanced photodegradation of the analyzed dyes, and on the other hand, suppress the recombination of photogenerated electrons and holes favoring the adsorption of dyes molecules. Thin layers of BBFO2 can then decompose pollutants at a rate that can exceed 50%, which is a very acceptable rate compared to that obtained in many published works [47,55,56,57,58,59,60,61,62].

## Figures and Tables

**Table 1:** Structural (atomic) parameters obtained from the Rietveld refinement of the BBFO2 samples at room temperature.

Atom	Atom Position			Bond Angle/Bond Length	
	x	y	z		
Bi/Ba	0.00000	0.00000	0.00000	O–Fe–O	78.856°
Fe	0.00000	0.00000	0.22701	O–Bi/Ba–O	116.671°
O	0.46262	0.05599	0.96664	Bi/Ba–O	2.4825 Å
				Fe–O	1.8937 Å

**Table 2:** Diffractometer data collection parameters, crystal structure parameters, and reliability factors of BBFO2 thin films obtained from Rietveld refinement at room temperature.

Molecular formula	Bi_0.98_Ba_0.02_FeO_3_
Diffractometer	PANalytical Almelo
CuKa radiation	λ = 1.5405 Å
Scan mode	θ–2θ
2 h range	20–70°
Scan width-scan speed	0.02, 2° min^−1^
Crystal system	Trigonal
Space group	*R3c*
Unit cell parameters	a = b = 5.579 Å c = 13.739 Å
	α = β = 90° γ = 120°
Volume	370.33818 Å^3^
Crystallite size	14.55 nm
Tolerance factor	0.892
Lattice strain	0.554 (%)
Profile function	Pseudo-Voigt
FWHM parameters (U, V and W)	−0.244962, 0.109213, −0.166533
Pattern residual (R_p_)	33
Weighted pattern residual (R_wp_)	34.8
Expected residual (R_exp_)	10.88
Bragg factor (R_B_)	19.25
Structural factor (R_F_)	12.8
Goodness (χ2)	10.3

## Data Availability

The original contributions presented in this study are included in the article. Further inquiries can be directed to the corresponding author.
